# Shifts in Soil Fungal Community and Trophic Modes During Mangrove Ecosystem Restoration

**DOI:** 10.3390/jof11020146

**Published:** 2025-02-14

**Authors:** Xiaofang Shi, Shengyao Zhou, Lanzi Xu, Rajapakshalage Thashikala Nethmini, Yu Zhang, Liangliang Huang, Ke Dong, Huaxian Zhao, Lianghao Pan

**Affiliations:** 1 Guangxi Key Lab of Mangrove Conservation and Utilization, Guangxi Academy of Marine Sciences (Guangxi Mangrove Research Center), Guangxi Academy of Sciences, Beihai 536000, China; shixiaofang_06@163.com; 2 Key Laboratory of Climate, Resources and Environment in Continental Shelf Sea and Deep Sea of Department of Education of Guangdong Province, Department of Oceanography, Key Laboratory for Coastal Ocean Variation and Disaster Prediction, College of Ocean and Meteorology, Guangdong Ocean University, Zhanjiang 524000, China; syzhou001009@163.com (S.Z.); yaya942636@163.com (L.X.); nethmini1207@gmail.com (R.T.N.); 3 College of Environmental Science and Engineering, Guilin University of Technology, Guilin 541004, China; llhuang@glut.edu.cn; 4 School of General Education, Guangxi Vocational University of Agriculture, Nanning 530003, China; yz1332125778@126.com; 5 Department of Biological Sciences, Kyonggi University, 154-42, Gwanggyosan-ro, Yeongtong-gu, Suwon-si 16227, Gyeonggi-do, Republic of Korea; dongke-007@163.com; 6 Key Laboratory of Ministry of Education for Environment Change and Resources Use in Beibu Gulf, Guangxi Key Laboratory of Earth Surface Processes and Intelligent Simulation, Nanning Normal University, Nanning 530001, China

**Keywords:** mangrove, restoration, soil fungi, fungal community, trophic mode, environmental effects, bioindicator

## Abstract

Mangrove ecosystems are valuable coastal ecosystems; however, studies on the diversity and functional features of their soil fungal communities during restoration are limited. In this study, we examined fungal diversity and trophic modes across mudflat, young mangrove, and mature mangrove stages. We found that Ascomycota and Basidiomycota were the dominant phyla, with saprotrophs as the most abundant trophic mode. The abundance of the major phyla and trophic modes significantly varied across restoration stages. Although fungal alpha (α)-diversity remained stable among the stages, beta (β)-diversity showed significant differentiation. Spearman’s analysis and partial Mantel tests revealed that total nitrogen and inorganic phosphorus significantly influenced the fungal α-diversity, whereas temperature and pH primarily shaped the fungal β-diversity. Total nitrogen and carbon were key factors affecting the trophic mode α-diversity, whereas total phosphorus and inorganic phosphorus were the main drivers of the trophic mode β-diversity. Variation partitioning analysis confirmed that nutrients, rather than soil properties, were the primary factors shaping fungal communities and trophic modes. Random forest analysis identified key bioindicators, including species such as Paraphyton cookei, and trophic modes such as saprotrophs, both of which were strongly influenced by soil carbon. These findings advance our understanding of fungal ecology in mangrove restoration.

## 1. Introduction

Soil microbiomes represent one of the most biodiverse communities on Earth, comprising approximately one-quarter of the total biodiversity [1,2,3]. These microorganisms form a vast and diverse network in soils, typically containing over 1000 kg of microbial biomass carbon per hectare [3]. They are integral to ecosystem functioning and influence critical processes, such as nutrient cycling, soil fertility, and carbon sequestration. For example, soil microbial diversity supports essential functions, such as organic matter decomposition and nutrient mineralization, that promote plant growth and ecosystem stability [4,5]. Heterogeneity in soil physicochemical properties, climate change, and human activity can alter the structure and function of soil microbial communities. For example, soil pH is a major determinant, with acidophilic and alkaline taxa displaying distinct preferences [2,6]. Temperature, water content, and other soil physicochemical properties are important drivers of microbial diversity [6,7]. Evidence suggests that the availability of soil nutrients, such as nitrogen and phosphorus, strongly influences the community structure, as microbes adjust their metabolic pathways to utilize available resources [6,8]. Human activities can alter the climate, which in turn changes the vegetation diversity and soil properties, thereby affecting soil microbial communities. Urban land use and pollution can also directly affect soil microbial composition and function [6,9,10,11,12]. However, the direct and indirect effects of environmental changes on microbial structure and function remain largely unknown.

Fungal communities are integral to soil ecosystems and act as key regulators of energy flow and nutrient transformation [10,13,14]. They play pivotal roles in organic matter decomposition and significantly influence ecosystem stability and productivity [10,13,14]. The factors driving soil fungal communities in terrestrial ecosystems have been extensively studied. For instance, in European coniferous forests, soil physical and chemical characteristics, such as the carbon to nitrogen (C:N) ratio and pH, significantly influence fungal community structure [15]. Soil fungal communities in the grasslands of the Loess Plateau are primarily shaped by factors such as ammonium-nitrogen (NH_4_^+^-N), water content, and dissolved organic carbon [13]. Total carbon is a critical factor influencing fungal community structure in boreal forests [16]. In current ecological research, linking specific soil microbial processes to particular microbial taxa is challenging [2]. Recent studies have shown that soil fungi can be classified into various types based on their survival strategies, such as saprotrophic, symbiotrophic, and pathotrophic [17]. Environmental changes not only affect fungal communities but may also influence their trophic modes. For instance, total carbon and nitrogen are key factors that shape the trophic modes of soil fungi in boreal forests [16]. In semi-arid grasslands in northern China, the addition of nitrogen and phosphorus significantly affected the diversity of pathogens and symbiotic fungi, whereas saprotrophic fungal diversity was primarily influenced by the addition of nitrogen [18]. Nickel contamination can alter the abundance of symbiotic fungi in agricultural soils [10]. However, the fungal diversity and trophic modes in coastal interface ecosystems remain unclear.

Mangroves are classic examples of coastal interface ecosystems, renowned for their distinctive intertidal features and important ecological roles [19]. They are vital for coastal protection and carbon sequestration but are under severe threat from global climate change and anthropogenic activities, including deforestation and urbanization [20,21,22]. Mangrove soil microbiomes play a critical role in maintaining biogeochemical cycles, supporting ecosystem stability in complex environments, facilitating material turnover, and linking terrestrial and marine ecosystems [23]. Several studies on fungal communities in mangrove sediments have been conducted based on high-throughput sequencing [24,25,26]. For example, research in New Caledonia in the South Pacific, southeastern China’s mangroves, and Sanya, China has revealed the dominance of Ascomycota and Basidiomycota. The structure of mangrove soil fungal communities in Sanya was primarily influenced by factors such as salinity, total organic carbon, and NH_4_^+^-N [26]. Recent studies have explored the differentiation of fungal community structures and functions during mangrove succession. Sun et al. [22] found that during the succession of Sonneratia apetala in southern China, Ascomycota and Basidiomycota were the dominant fungal phyla. Environmental factors, including effective potassium, pH, total nitrogen, NH_4_^+^-N, and available phosphorus, significantly influenced fungal community structure. The abundance of dung saprotrophs and undefined fungi varies significantly across different restoration stages [22]. Another study found that in mangroves formed by the conversion of receding ponds, Ascomycota was the dominant fungal phylum, fungal diversity increased, and soil saprotrophs decreased in naturally recovered mangroves [27]. However, the differentiation of fungal communities and their trophic modes across different habitats during mangrove restoration remains unclear.

Therefore, we conducted a study in the Beilun River Estuary in Dongxing City, Guangxi, within a mangrove reserve. This reserve encompasses three distinct habitats, namely mudflats, young mangroves, and mature mangroves, representing the natural successional stages from mudflats to mature mangroves. By analyzing mangrove sediment samples, we aimed to investigate the changes in fungal functions throughout the restoration and succession processes; specifically, we aimed to: (1) examine the differentiation of fungal diversity and trophic modes across different restoration and succession stages; (2) identify the key factors driving these fungal trophic modes; and (3) analyze bioindicators at different restoration stages. This study will enhance our understanding of the mechanisms that maintain fungal community diversity and functional features during mangrove restoration and provide scientific support for effective restoration and management.

## 2. Materials and Methods

### 2.1. Study Sites and Sampling Design

This study was conducted in the Beilun River Estuary in Dongxing City, Guangxi, within a mangrove restoration area (Figure S1). The restoration system encompassed three distinct habitats, namely, mudflats (MFs), young mangroves (YMs), and mature mangroves (MMs), representing the natural succession stages from mudflats to young and mature mangroves (Figure S1). MFs are characterized by open, waterlogged soil with no vegetation. YMs comprise newly established mangrove saplings and a small number of salt marsh plants. MMs feature well-developed trees. Sediment samples were collected from these habitats on 26 December 2021. Three plots were established for sampling in each habitat. Samples were collected from three depths using a three-point sampling method: surface (S; 0–5 cm), middle (M; 10–15 cm), and bottom (B; 20–25 cm). A total of 500 g of sediment was collected and homogenized at each depth. A total of 81 sediment samples were collected.

### 2.2. Physical and Chemical Analytical Procedures

Biochemical parameters were assessed for each sample (Figure S2). During sampling, we employed a portable salinity tester (PNT3000, STEP Systems GmbH, Nuremburg, Germany) to measure the salinity of the sediment pore water. Sediment pH and temperature were recorded using a HI98121 tester (Hanna instruments, Woonsocket, RI, USA). Water content was determined by the weight loss of wet sediment after drying at 85 °C for 24 h. Total nitrogen (TN), total carbon (TC), total organic carbon (TOC), and total inorganic carbon (TIC) were determined using an elemental analyzer (Vario MacroCube, Elementar, Langenselbold, Germany). Total phosphorus (TP) and dissolved phosphate (PO_4_^3−^) were quantified using molybdenum blue colorimetry and flame photometry. Inorganic (Pi) and organic phosphorus (Po) were determined using the method described by DeLuca et al. [22]. For the measurement of inorganic nitrogen in sediment (NH_4_^+^-N, NO_3_^−^-N, and NO_2_^−^-N), each sample was extracted using a 2 M KCl solution at a soil-to-solution ratio of 1:4, shaken for 1 h at 200 rpm, and analyzed using a continuous flow analyzer (SAN++, Skalar, Breda, The Netherlands). Water content was gravimetrically determined by weighing the sediment samples before and after drying at 105 °C for 24 h.

### 2.3. DNA Extraction, Gene Amplification, and Sequencing

DNA was extracted from the samples using a PowerSoil DNA Isolation Kit (QIAGEN, Hilden, Germany) following the manufacturer’s protocol. The purity and concentration of DNA were determined with a Thermo Scientific NanoDrop1000 spectrophotometer (Thermo Fisher Scientific, Wilmington, DE, USA) and stored at −80 °C. The fungal internal transcribed spacer 1 (ITS1) region was amplified by polymerase chain reaction (PCR) using the dual-bar coded primers ITS1F (5′-CTTGGTCATTTAGAGGAAGTAA-3′) and ITS2 (5′-GCTGCGTTCTTCATCGATGC-3′). Each 50 μL PCR reaction was conducted according to standard procedures for 35 cycles, with a denaturation step at 95 °C for 30 s, annealing at 55 °C for 30 s, and extension at 72 °C for 45 s. The resulting PCR products were prepared using a TruSeq DNA kit (Illumina, San Diego, CA, USA) according to the manufacturer’s instructions. The purified library was prepared following the Illumina library preparation protocols and sent to Shanghai Majorbio Bio-Pharm Technology Co., Ltd. (Shanghai, China) for paired-end sequencing on an Illumina MiSeq platform (PE250, 2 × 250 bp, Illumina, San Diego, CA, USA). All the sequence data were deposited in GenBank under the BioProject Accession PRJNA1213986.

### 2.4. Bioinformatics and FUNGuild

The raw ITS2 sequencing data were processed using QIIME2 software (version 2020.2). The DADA2 plugin was used for quality control, denoising, chimera removal, and the generation of amplicon sequence variants (ASVs). Taxonomic annotation of fungal sequences was conducted using the Ribosomal Database Project (RDP) classifier in conjunction with a specific fungal ITS1 sequence database built by the National Center for Biotechnology Information (NCBI). For downstream analysis, only sequences classified within the kingdom of fungi were considered. The functional groups of fungi were determined using FUNGuild v1.0, namely, a flat database available on https://github.com/UMNFuN/FUNGuild (accessed on 18 March 2022). To avoid over-interpretation of fungal functional groups, we excluded entries with a confidence level of “possible” and retained only those classified as “highly probable” or “probable”. Fungal communities that could not be identified—or were identified with multiple complex nutritional methods—were categorized as “undefined”.

### 2.5. Statistical Analyses

To minimize the bias associated with sequencing coverage and enable the comparison of community patterns among samples, we normalized the number of sequences in each sample to match the smallest sample size. This resampling was conducted using the “rrarefy” function from the R package “vegan” before conducting the statistical analyses.

The majority of the statistical analyses were conducted in R, utilizing packages such as “vegan”, “picante”, “plspm”, “ggplot2”, and “Hmisc”. Differences in alpha (α)-diversity and environmental parameters across different environments were assessed using a one-way analysis of variance (ANOVA). The Spearman’s rank correlation method was used to calculate the correlations. Indices such as species richness, Shannon index, Simpson index, Chao1 index, and Good’s coverage were calculated. In particular, the Shannon index was used to represent α-diversity, whereas the richness index indicated the species pool size. Beta (β)-diversity was assessed using the Bray–Curtis distance, and the similarity analysis (PerMANOVA) among different groups was conducted using the “adonis” function from the “vegan” package.

A partial Mantel test, with 9999 permutations, was conducted using the “mantel.partial” function in the “vegan” package. A variation partitioning analysis (VPA) was conducted using the “varpart” function in the “vegan” package.

## 3. Results

### 3.1. Soil Fungal Diversity and Community Composition

**Figure 1:**
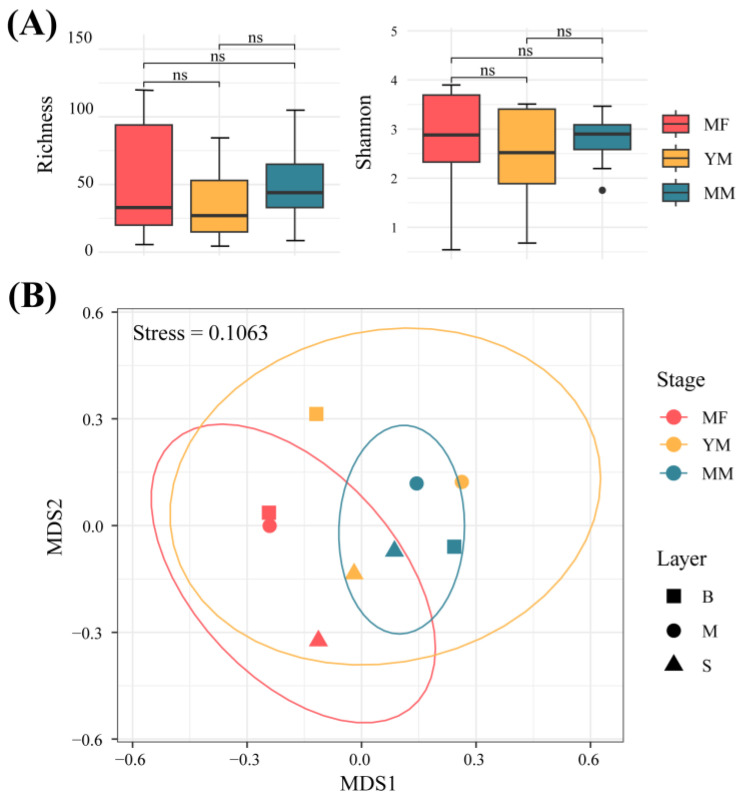
Alpha (α)- and beta (β)-diversities across the three restoration stages. (**A**) α-diversity (richness and Shannon index), with the median indicated by a horizontal line. (**B**) Non-metric multidimensional scaling (NMDS) plot based on the Bray–Curtis dissimilarity of fungal communities, wherein gray lines represent the within-group variability. MF: mudflat; YM: young mangrove; MM: mature mangrove.

A total of 359,632 sequence reads representing 687 fungal ASVs were retained in the fungal ITS dataset after quality filtering and discarding the rare ASVs. Good’s coverage was >99.91% (Table S1) for each sample, indicating that most fungal taxa were successfully recovered. Fungal ASVs were predominantly classified as Ascomycota (56.69%), followed by Basidiomycota (33.07%), Mucoromycota (5.91%), and Zoopagomycota (1.26%) (Figures S3 and S4). The relative abundance of Ascomycota was significantly higher in the YM stage (74.31%) than in both the MF (50.03%) and MM (59.62%) stages (*p* < 0.05) (Figure S4). No significant difference was observed in the abundance of Ascomycota between the MF and MM stages. In contrast, the relative abundance of Basidiomycota was significantly higher at the MF stage (42.68%) than at the YM stage (22.89%) (*p* < 0.05), whereas no significant differences were observed between the YM and MM stages (31.05%) or MM and MF stages (Figure S4). We assessed the α-diversity of fungal communities using both the richness and Shannon indices. No significant differences were observed in either the richness or Shannon index across the different restoration stages (Figure 1A). In contrast to the α-diversity, both analyses of similarities ((ANOSIM) (*p* < 0.001) and PerMANOVA (*p* < 0.001) revealed significant differences in fungal β-diversity among the three restoration stages at the ASV level (Table S2). Non-metric multidimensional scaling (NMDS) analysis based on Bray–Curtis dissimilarities further indicated a gradual shift in the fungal community structure throughout mangrove restoration, with the MF and MM samples being clearly separated, whereas the YM samples exhibited partial overlap with both the MF and MM samples (Figure 1B).

### 3.2. Trophic Mode Features of the Soil Fungal Community

**Figure 2:**
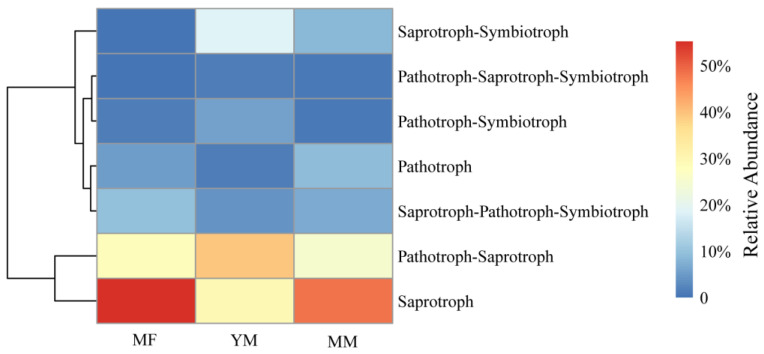
Relative abundance distribution of fungal trophic modes across different stages of mangrove restoration in this study.

In this study, fungal taxa were categorized into seven trophic modes (saprotroph, pathotroph, pathotroph–saprotroph, saprotroph–symbiotroph, pathotroph–symbiotroph, pathotroph–saprotroph–symbiotroph, and saprotroph–pathotroph–symbiotroph) and 14 guilds using the FUNGuild database (Figure 2 and Figure S5). Saprotrophs were the most predominant trophic mode, accounting for 41.62% of all the observed trophic modes (Figure 2), primarily comprising dung saprotroph–plant saprotroph–wood saprotroph and wood saprotroph guilds (Figure S5). The second most abundant trophic mode was pathotrophic-saprotrophs (34.59%) (Figure 2), primarily comprising an animal pathogen–undefined saprotroph guild (Figure S5). The top five trophic modes were saprotroph–symbiotroph (8.91%), saprotroph–pathotroph–symbiotroph (6.69%), and pathotroph–saprotroph–symbiotroph (0.62%) (Figure 2). No symbiotrophs were found in the single trophic mode, and only pathotrophs were observed, accounting for 5.26% of the fungal taxa (Figure 2). These primarily comprised plant and animal pathogen guilds (Figure S5). Significant differences (*p* < 0.05) in trophic mode abundance were observed across regions, except for the pathotroph–symbiotroph (Figure 2). The highest abundance of saprotrophs was found in the MF area, whereas pathotroph–saprotrophs peaked in the YM zone (Figure 2).

The ASV α-diversity of the two predominant trophic modes, namely, saprotrophs and pathotroph–saprotrophs, indicated no significant differences across regions, except for the richness of saprotrophs, which differed significantly between the MF and YM regions (Figure S6).

Both ANOSIM (*p* < 0.001) and PerMANOVA (*p* < 0.001) revealed significant differences in the composition of fungal trophic modes across regions (Table 1). No significant differences were observed between the MF and MM groups (Table 1). Significant differences were observed between the YM and the other two regions (Table 1), indicating that the fungal trophic mode composition significantly changed from the MF to YM stages, and from the YM to MM stages.

### 3.3. Environmental Effects on the Fungal Community and Trophic Modes

We examined the Spearman’s correlations between the fungal community α-diversity and environmental factors (Table S3). Overall, TN and Pi were the most significant factors influencing both richness and the Shannon index (*p* < 0.001) (Table S3). However, the key factors varied across the different restoration stages (Table S3). In the MF zone, temperature was the most significant factor (Table S3). TN and TOC were the predominant factors in the YM zone (Table S3). In contrast, TN and salinity were the most influential factors in the MM zone (Table S3).

Overall, considering the α-diversity of the trophic modes, Spearman’s correlation analysis revealed that TN was the most influential factor affecting richness, whereas TC had the strongest impact on the Shannon index (Table S4). In the MF zone, Pi was the most significant factor influencing both richness and the Shannon index (Table S4). In the YM zone, TOC was the predominant factor affecting both indices (Table S4). In contrast, in the MM zone, NO_2_^−^-N was the main factor affecting richness, whereas no significant correlations were found between the Shannon index and environmental factors (Table S4).

**Figure 3:**
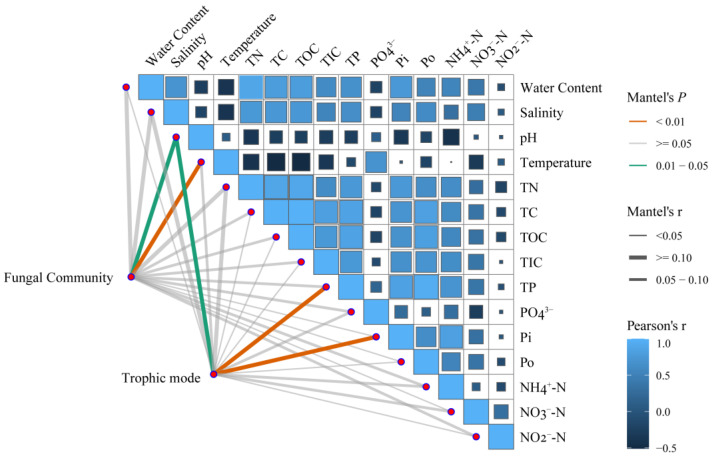
Pairwise correlations between environmental factors (top right) and partial Mantel tests for fungal communities and trophic modes with each environmental factor. TN: total nitrogen; TC: total carbon; TOC: total organic carbon; TIC: total inorganic carbon; TP: total phosphorus; PO_4_^3^⁻: dissolved phosphate; Pi: inorganic phosphorus; Po: organic phosphorus.

Partial Mantel tests were conducted to examine the correlation between environmental factors and β-diversity in soil fungal communities (Figure 3 and Table S5). Overall, temperature and pH were the key factors influencing the β-diversity of fungal communities (Figure 3 and Table S5). In the MF zone, TN was the most influential factor (Table S5). In the YM zone, pH was the predominant factor (Table S5). In the MM zone, NO_2_^−^-N and PO_4_^3^⁻ were the most significant factors (Table S5).

Overall, TP was the most influential factor for the β-diversity of the trophic modes (Figure 3 and Table S6). TP was the most significant factor in the MF zone (Table S6). In both the YM and MM zones, NO_2_^−^-N was identified as the most influential factor (Table S6).

**Figure 4:**
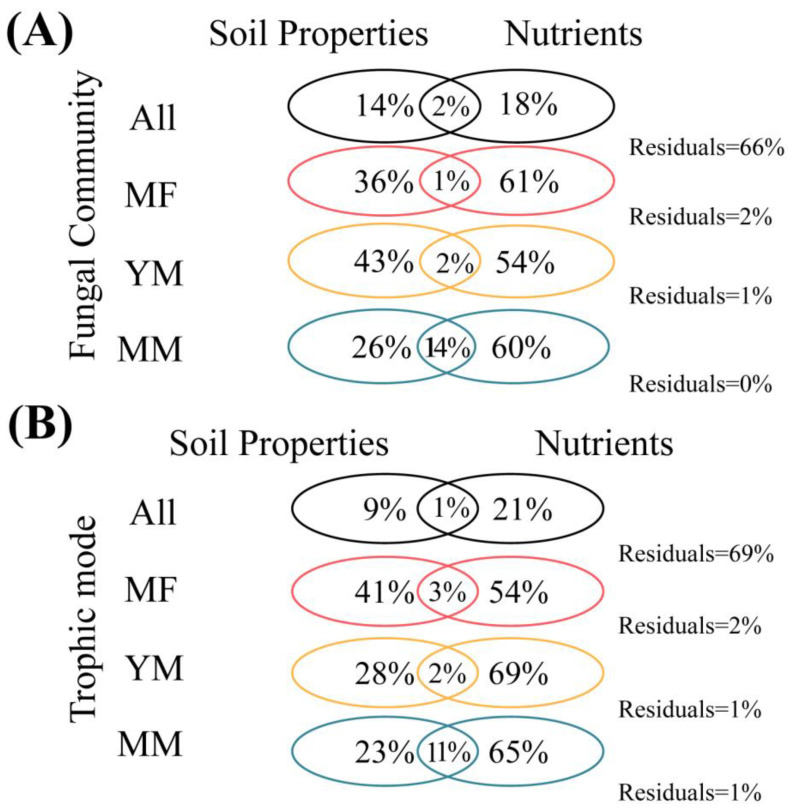
Variation partitioning analysis of the effects of soil properties (water content, salinity, pH, and temperature) and nutrients (TN, TC, TOC, TIC, TP, PO_4_^3^⁻, Pi, Po, NH_4_^+^-N, NO_3_^−^-N, and NO_2_^−^-N) in the fungal community (**A**) and trophic mode composition (**B**).

We conducted variation partitioning analysis (VPA) to assess the contributions of soil physicochemical parameters (i.e., water content, salinity, pH, and temperature) and nutrient variables (i.e., TN, TC, TOC, TIC, TP, PO_4_^3^⁻, Pi, Po, NH_4_^+^-N, NO_3_^−^-N, and NO_2_^−^-N) to the fungal community and trophic mode composition (Figure 4). Our results revealed that both overall and across different regions, the pure effects of nutrients on the fungal community structure and trophic mode composition were greater than those of soil physicochemical parameters (Figure 4). In particular, for the fungal community structure, the pure effect of nutrients was the largest in the MF zone (61%) (Figure 4). The effect of nutrients on the fungal trophic mode composition was most pronounced in the YM zone (69%) (Figure 4).

### 3.4. Bioindicators in Different Successional Environments

**Figure 5:**
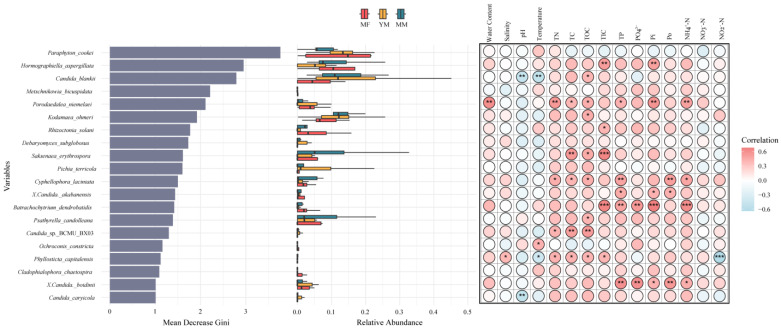
Bioindicators of fungal species in different mangrove restoration stages. **Left**: Gini rank of the bioindicator species, representing their importance level; **Middle**: Relative abundances of the bioindicator species; **Right**: Spearman’s correlations between the relative abundances of the species and environmental and nutrient factors. The asterisk indicates the significance level: *, *p* < 0.05; **, *p* < 0.01; ***, *p* < 0.001.

We employed a random forest approach to analyze the bioindicators across different restoration stages. Among the fungal species, *Paraphyton cookei*, *Hormographiella aspergillata*, *Candida blankii*, and *Metschnikowia bicuspidata* exhibited the highest Gini values, indicating that they were the most significant bioindicator species (Figure 5). Their abundances showed significant differences across the restoration stages (Figure 5). Spearman’s correlation analysis further revealed that the abundance of most of the top 20 species was significantly correlated with various environmental factors (Figure 5). For example, *Hormographiella aspergillata* was positively correlated with TIC and Pi, whereas *Candida blankii* was significantly negatively correlated with pH and temperature (Figure 5). Among the top 20 species, TOC was significantly correlated with eight species (*p* < 0.05), making it the most frequent influencing factor (Figure 5).

**Figure 6:**
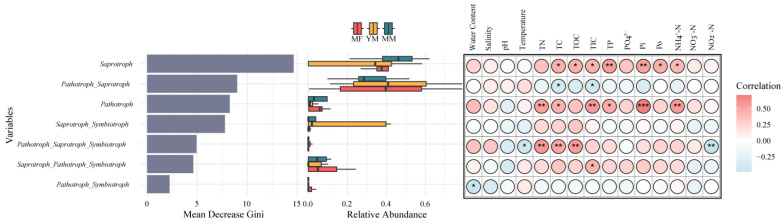
Bioindicators of fungal trophic modes in different mangrove restoration stages. **Left**: Gini rank of the bioindicator trophic modes, representing their importance level; **Middle**: relative abundances of the bioindicator trophic modes; **Right**: Spearman’s correlations between the relative abundances of the trophic modes and environmental and nutrient factors. The asterisk indicates the significance level: *, *p* < 0.05; **, *p* < 0.01; ***, *p* < 0.001.

Among the trophic modes, saprotrophs, pathotroph–saprotrophs, and pathotrophs exhibited the highest Gini values (Figure 6), indicating that they were the most effective indicators of environmental change across the restoration stages. Their abundances varied significantly across the stages (Figure 2 and Figure 6). With the exception of the saprotroph–symbiotroph mode, the abundance of all trophic modes was significantly influenced by certain environmental factors (Figure 6). In particular, saprotrophs were significantly affected by TC, TOC, TIC, TP, Pi, Po, and NH_4_^+^-N (*p* < 0.05) (Figure 6). Both the TC and TIC were the most frequent factors affecting the abundance of the four trophic modes (Figure 6).

## 4. Discussion

Fungal communities play crucial roles in nutrient cycling and ecosystem functioning in mangrove environments [20,22,24]. However, our understanding of the fungal community features during the restoration of these ecosystems remains limited. In this study, we investigated the responses of fungal community diversity and trophic modes to environmental changes during different stages of anthropogenic mangrove restoration.

Consistent with the results of previous studies [22,24] on mangrove sediments, we identified Ascomycota and Basidiomycota as the dominant fungal phyla. In particular, Ascomycota has been widely recognized as the predominant fungal group in mangrove ecosystems [28]. A global comparative analysis of soil fungi revealed that individuals of the Ascomycota phylum possess a diverse array of genes associated with nutrient acquisition, such as phosphate transporters, nitrogen immobilization, and carbohydrate metabolism [29]. Similarly, Basidiomycota also plays a significant role in nutrient cycling, particularly in lignin degradation and organic matter decomposition [29,30]. These functional traits likely contribute to the dominance of both fungal phyla in mangrove environments. A previous study of the soil fungal community of *Sonneratia apetala* mangroves revealed that Ascomycota and Basidiomycota were the dominant fungal phyla, but their abundance did not vary significantly with plantation age [22]. In contrast, the mangrove communities in this study, which predominantly comprised *Avicennia marina* and *Kandelia obovata*, exhibited significant variation in the abundance of Ascomycota and Basidiomycota across different restoration stages. In particular, the abundance of Ascomycota peaked at the YM stage, whereas that of Basidiomycota peaked at the MM stage. These differences suggest that the tree species composition plays a critical role in shaping the fungal community structure, likely owing to variations in root exudates, litter input, and microhabitat conditions associated with different plant species [31,32].

We found no significant differences in the α-diversity (i.e., richness and Shannon indices) of fungal communities between the different recovery stages (Figure 1A). However, the β-diversity showed significant differences (Figure 1B). This is consistent with the results of previous studies on *Sonneratia apetala* at different plantation ages [22]. These results suggest that soil fungal α-diversity did not vary significantly during the restoration period, whereas notable changes were observed in β-diversity [22]. A previous study on coastal fungi also suggested that the response of planktonic fungi to environmental variation was more pronounced in β-diversity than in α-diversity [33]. These findings indicate that although the overall richness and evenness of mangrove soil fungal communities remain relatively stable during ecological restoration, their specific composition is highly responsive to environmental changes and successional stages. This might be because fungi undergo adaptive adjustments at different recovery stages in response to varying environmental conditions [34]. Fungi with stronger adaptive capacities can undergo rapid proliferation even with small changes in taxonomic diversity, leading to greater community turnover [34]. We also examined the impact of environmental factors on fungal diversity and found that TN and Pi are key determinants of overall soil fungal α-diversity (*p* < 0.001) (Table S3). TN was also identified as a critical factor influencing α-diversity in mangrove soils (YM and MM) (*p* < 0.01) but had no effect in MF soils (Table S3). Mangrove ecosystems are characterized by significant nitrogen fixation owing to high organic matter input, anaerobic conditions, and microbial activity [35]. Ongoing nitrogen input, potentially influenced by human activities, such as agricultural runoff and sewage discharge, and phosphorus loss lead to phosphorus limitation, which may affect the fungal diversity [36,37]. Partial Mantel tests indicated that temperature and pH were the key drivers of soil fungal β-diversity in our study area (Figure 3).

Previous studies on *Sonneratia apetala* plantations of varying ages and mangrove ecosystems converted from ponds have indicated that pH is a critical factor in shaping the soil fungal communities in successive mangrove habitats [22,27]. However, these studies did not consider the soil temperature [22,27]. The observed influence of temperature is understandable as the presence of plants can affect sunlight exposure. In our study, vegetated areas had lower soil temperatures (Figure S2). Continental-scale research on forest soil fungal diversity has indicated that temperature and pH are universal factors shaping the fungal community structure [38], which aligns with and supports our findings. Additionally, we found that the primary factors influencing the fungal community structure varied across different stages of restoration (Table S5), reflecting the habitat-dependent nature of the environmental effects on microbial communities [39]. The environmental impact on the soil fungal community may not be independent of individual factors; therefore, we constructed VPA to explain the effects of soil properties and nutrients [40]. Our study suggests that fungal communities in coastal environments are strongly influenced by the nutrient content, which can be altered by human activities, such as agriculture or pollution.

Fungi employ various mechanisms for nutrient acquisition, leading to distinct trophic modes and functional diversities under different environmental conditions [16,41,42,43]. Our findings indicate that saprotrophs are the predominant trophic mode in fungal communities and that other highly abundant trophic modes also incorporate saprotrophic characteristics (Figure 2). The prominence of saprotrophs may be attributed to their crucial role in decomposition, which helps sustain nutrient cycling and carbon storage [22,27,44,45]. These fungi are prevalent in mangrove environments [22,27] and other marine ecosystems, including surface waters, intertidal zones, and deep-sea sediments [44,45]. Consistent with the results of previous studies [22,27], we found that soil saprotrophs were less abundant in successional mangrove environments than in mudflats or shrimp ponds (Figure 2). Saprotroph abundance was the lowest in the YM zone (Figure 2). Although mangrove soils are rich in organic matter [46,47], which leads to higher saprotroph abundance, vegetation characteristics also influence fungal function [43,48]. Consequently, other trophic modes (e.g., pathogens and symbiotrophic fungi) may occupy ecological niches, particularly in young mangrove zones where mangroves are still developing, leading to a reduction in the relative abundance of saprotrophs. Notably, this does not imply that the absolute abundance of saprotrophs in mangrove environments is lower than that in mudflats. Similar to fungal α-diversity, the α-diversity of the dominant trophic modes, namely, saprotrophs and pathotroph–saprotrophs (except for saprotroph richness), indicated no significant differences across the three environments (Figure S6), suggesting that these fungal groups may adapt to the environment through community turnover rather than through changes in taxonomic diversity [49].

Considering the trophic modes of the fungal communities, we found no significant differences between the MF and MM zones, but both were significantly different from those in the YM zone (Table 1). This may be attributed to the rapid growth and development of mangrove plants in the YM region, which induce considerable changes in soil properties through the input of leaf litter, root exudates, and other organic matter. Consequently, fungal trophic modes in this environment markedly differ from those in the relatively stable coastal sediments of MFs and MMs. Notably, we found that fungal nutrient acquisition strategies were responsive to variations in multiple soil factors. The α-diversity of trophic modes was primarily influenced by TN and TC, whereas β-diversity was mainly driven by TP and Pi (Figure 3). In particular, in different zones, C-, N-, and P-related factors exerted varying degrees of influence on trophic modes (Table S6), which is consistent with findings from other soil environments [16,49]. These results highlight the significant effect of soil nutrients on the functional composition of fungal communities. The VPA results further confirmed this effect, indicating that nutrient effects outweighed those of soil properties across all the samples and in the different zones (Figure 4B).

After confirming that soil fungal communities and their trophic modes were significantly influenced by environmental heterogeneity, we applied a random forest approach to identify bioindicators across different mangrove restoration stages. Our results revealed that fungi, such as *Paraphyton cookei*, *Hormographiella aspergillata*, *Candida blankii*, and *Metschnikowia bicuspidata*, serve as key indicator species of environmental changes during mangrove succession (Figure 5). Saprotrophs were identified as the most reliable trophic mode for indicating environmental change (Figure 6). A Spearman’s analysis also highlighted significant correlations between the abundance of these species and trophic mode with various environmental factors (Figure 5 and Figure 6). TOC was the most significant factor influencing the abundance of the top indicator species (Figure 5), supporting the view that significant changes in soil organic matter during mangrove succession affect the fungal community composition [50]. Consistent with this result, we observed a progressive increase in soil TOC along a mangrove succession gradient (Figure S2). Saprotrophs are the most abundant fungal group and serve as important bioindicators, which is unsurprising considering their crucial role in the decomposition of complex organic matter [50,51]. In mangrove environments during restoration, variations in soil chemistry and physical structure, resulting from changes in vegetation and land use, can significantly shape the composition of saprotrophic communities, offering diverse ecological niches, particularly with fluctuations in soil carbon levels [50,51,52]. We found that the abundance of saprotrophs was influenced by multiple soil factors, including carbon, nitrogen, and phosphorus (Figure 6), highlighting their sensitivity to changes in nutrient availability. Among all the trophic mode bioindicators, TC was the most widespread influencing factor (Figure 6). Similar to TOC, the TC content increased with mangrove succession (Figure S2). These results underscore the significant association between changes in carbon content during mangrove succession and fungal bioindicators, including species and trophic modes.

## 5. Conclusions

This study investigated the shifts in soil fungal communities and their trophic modes during mangrove ecosystem restoration, focusing on diversity patterns, environmental drivers, and bioindicators across different restoration stages. Fungal α-diversity (richness and Shannon index) remained stable across mudflat, young mangrove, and mature mangrove stages, whereas β-diversity exhibited significant turnover. Ascomycota and Basidiomycota were the most abundant phyla, with Ascomycota dominating young mangrove soils (74.3%) and Basidiomycota peaking in mudflats (42.7%). Total nitrogen and inorganic phosphorus were the primary drivers of α-diversity, while temperature and pH governed β-diversity. Nutrients explained the majority of fungal community variation in mangrove restoration soils. *Paraphyton cookei*, *Hormographiella aspergillata*, and saprotrophs emerged as key bioindicators, with their abundances strongly linked to soil carbon dynamics. Overall, this study contributes to our understanding of the complex interactions among fungal communities, environmental factors, and ecosystem functions in mangrove ecosystems, providing a foundation for the restoration and management strategies of mangroves.

## Figures and Tables

**Table 1:** ANOSIM and PerMANOVA tests of fungal community across different mangrove restoration stages. The top-left corner presents the overall results, whereas the table presents pairwise comparisons.

0.145 ***	MF	YM
MF		
YM	0.192 ***	
MM	0.004 ***	0.238 ***
0.141 ***	MF	YM
MF		
YM	0.129 ***	
MM	0.018 ***	0.153 ***

The upper left shows the overall test results, while the table presents the results of pairwise comparisons. Values in the ANOSIM results represent the r-statistic, while values in the PerMANOVA results represent the R^2^ statistic. The asterisk indicates the significance level: ***, *p* < 0.001. MF: mudflat; YM: young mangrove; MM: mature mangrove.

## Data Availability

All the sequence data were deposited in GenBank under the BioProject Accession PRJNA1213986.

## Supplementary Materials

The following supporting information can be downloaded at: https://www.mdpi.com/article/10.3390/jof11020146/s1, Figure S1: Sampling sites in the Beilun River Estuary mangrove restoration area, Dongxing City, Guangxi, China; Figure S2: Variation of soil environmental factors across different mangrove restoration areas; Figure S3: Community composition (phylum level) of soil fungi in this study; Figure S4: Community composition (phylum level) of soil fungi across different restoration stages; Figure S5: Relative abundance of functional guilds of the soil fungi; Figure S6: Alpha (α)-diversity of the most abundant trophic mode in mangrove restoration soils; Table S1: Alpha (α)-diversity index of the samples; Table S2. Results of ANOSIM and PerMANOVA tests for soil fungi community across different stages; Table S3: The Spearman’s correlations of the individual environmental factor to the fungal alpha (α)-diversity; Table S4: The Spearman’s correlations of the individual environmental factor to the alpha (α)-diversity of the fungal trophic modes; Table S5: The correlations of the partial Mantel test for individual environmental factor to the fungal community; Table S6: The correlations of the partial Mantel test for individual environmental factor to the fungal trophic modes.
