# Supplementary material for: Shifts in Soil Fungal Community and Trophic Modes During Mangrove Ecosystem Restoration
Source: J Fungi (Basel). 2025 Feb 14;11(2):146. doi: 10.3390/jof11020146 (PMC11856337; doi:10.3390/jof11020146)
Supplement: Supplementary file 1 [file jof-11-00146-s001.zip › Supplementary Information-1 (Figure S1-S6).pdf]

Shifts in Soil Fungal Community and Trophic Modes During Mangrove Ecosystem Restoration

Xiaofang Shi [1], Shengyao Zhou [2,3], Lanzi Xu [2], Rajapakshalage Thashikala Nethmini [2], Yu Zhang [4], Liangliang Huang [3], Ke Dong [5], Huaxian Zhao [6,*], and Lianghao Pan [1,*]

1 Guangxi Key Lab of Mangrove Conservation and Utilization, Guangxi Academy of Marine Sciences, Guangxi Mangrove Research Center, Guangxi Academy of Sciences, Beihai 536000, China; shixiaofang_06@163.com (X.S.); panlh86@163.com (L.P.)

2 Key Laboratory of Climate, Resources and Environment in Continental Shelf Sea and Deep Sea of Department of Education of Guangdong Province, Department of Oceanography, Key Laboratory for Coastal Ocean Variation and Disaster Prediction, College of Ocean and Meteorology, Guangdong Ocean University, Zhanjiang 524000, China; syzhou001009@163.com (S.Z.); yaya942636@163.com (L.X.); nethmini1207@gmail.com (R.T.N.)

3 College of Environmental Science and Engineering, Guilin University of Technology, Guilin 541004, China; llhuang@glut.edu.cn (L.H.)

4 School of General Education, Guangxi Vocational University of Agriculture, Nanning 530000, China; yz1332125778@126.com (Y.Z.)

5 Department of biological sciences, Kyonggi University, 154-42, Gwanggyosan-ro, Yeongtong-gu, Suwon-si, Gyeonggi-do 16227, Republic of Korea; dongke-007@163.com (K.D.)

6 Key Laboratory of Ministry of Education for Environment Change and Resources Use in Beibu Gulf, Guangxi Key Laboratory of Earth Surface Processes and Intelligent Simulation, Nanning Normal University, Nanning 530001, Guangxi, China; zhx343@gmail.com (H.Z.)

* Correspondence: zhx343@gmail.com (H.Z.); panlh86@163.com (L.P.)

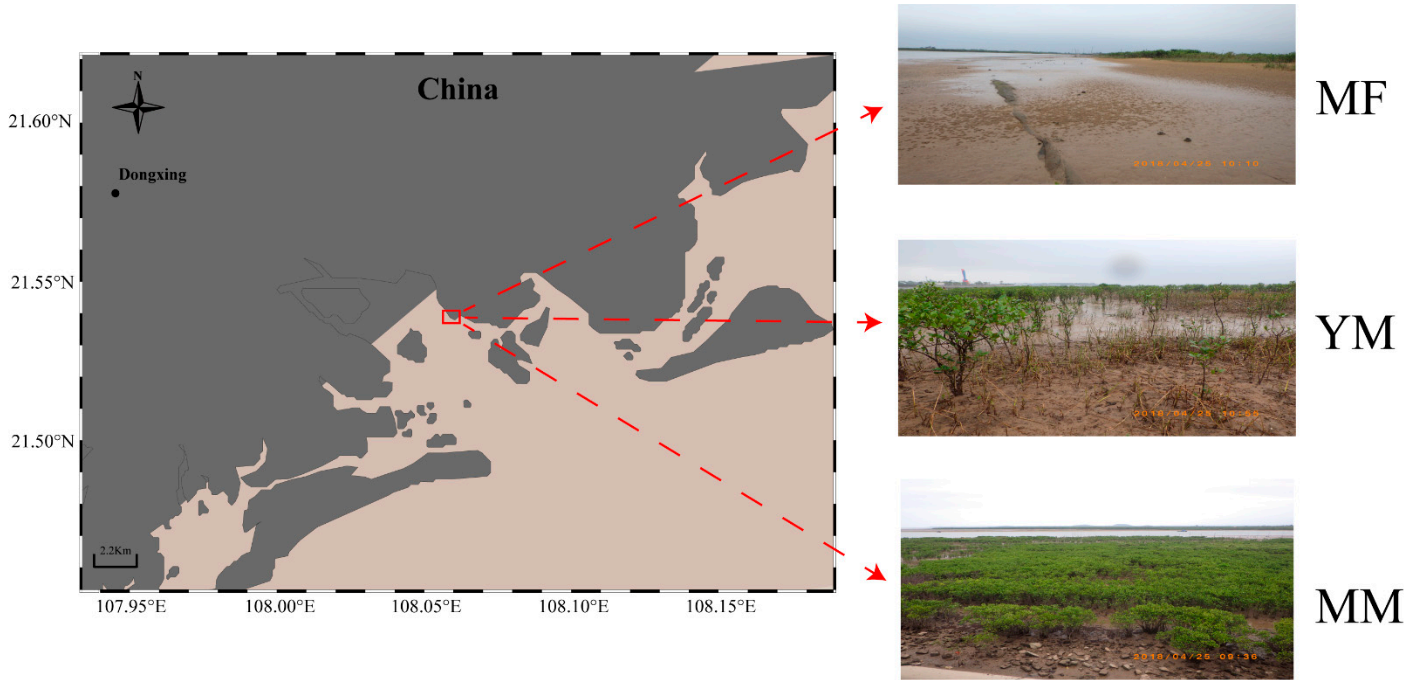

Figure S1. Sampling sites in the Beilun River estuary mangrove restoration area, Dongxing City, Guangxi, China. The study area includes three distinct habitats representing natural succession stages: mudflat (MF), young mangrove (YM), and mature mangrove (MM).

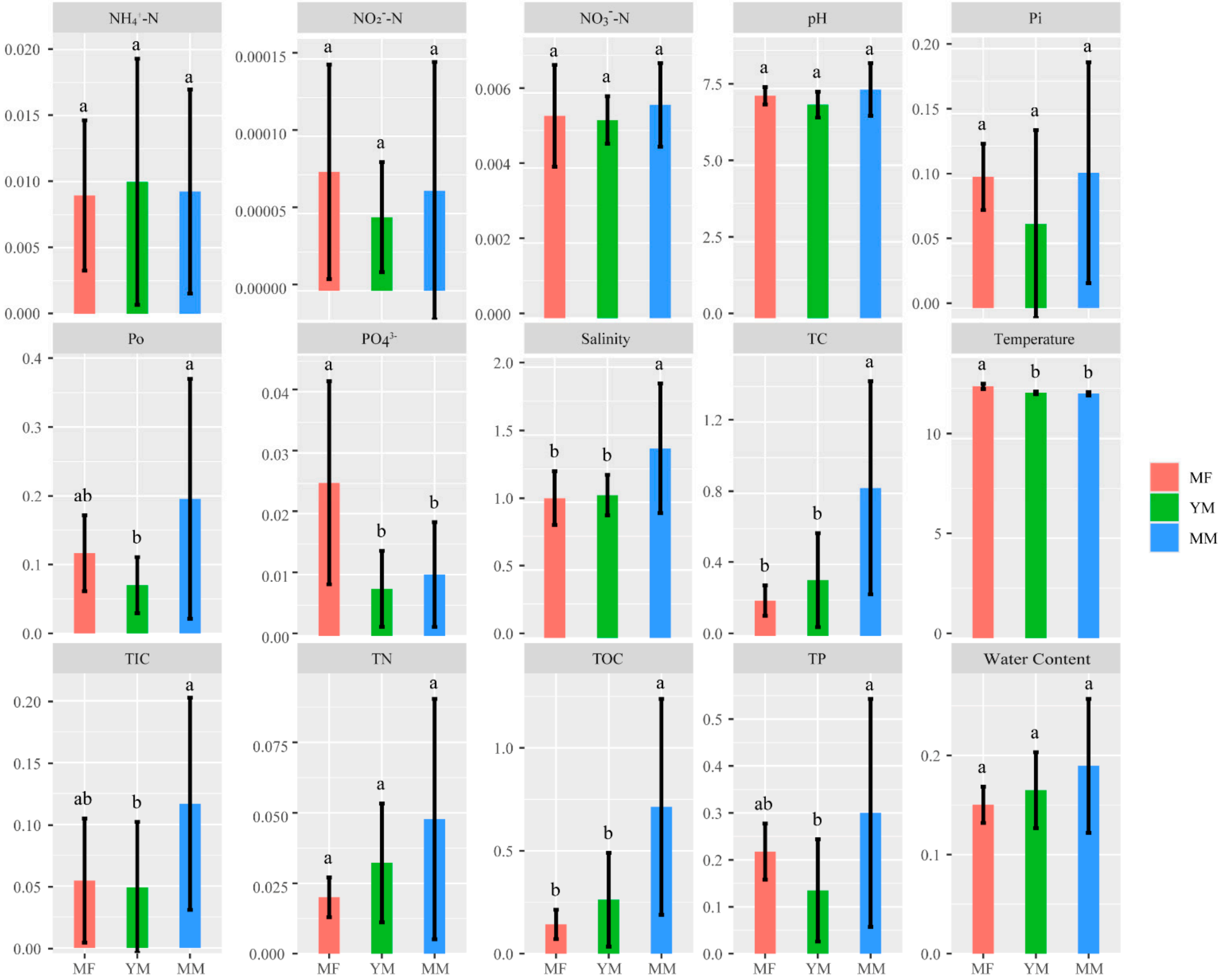

Figure S2. Variation of soil environmental factors across different mangrove restoration areas. MF: mudflat; YM: young mangrove; MM: mature mangrove; S: surface (0-5 cm depth); M: middle (10-15 cm depth); B: bottom (20-25 cm depth); TN: total nitrogen; TC: total carbon; TOC: total organic carbon; TIC: total inorganic carbon; TP: total phosphorus; PO_4^{3-}: dissolved phosphate; Pi: inorganic phosphorus; Po: organic phosphorus; NH_4^+-N: ammonium nitrogen; NO_3^--N: nitrate nitrogen; NO_2^--N: nitrite nitrogen.

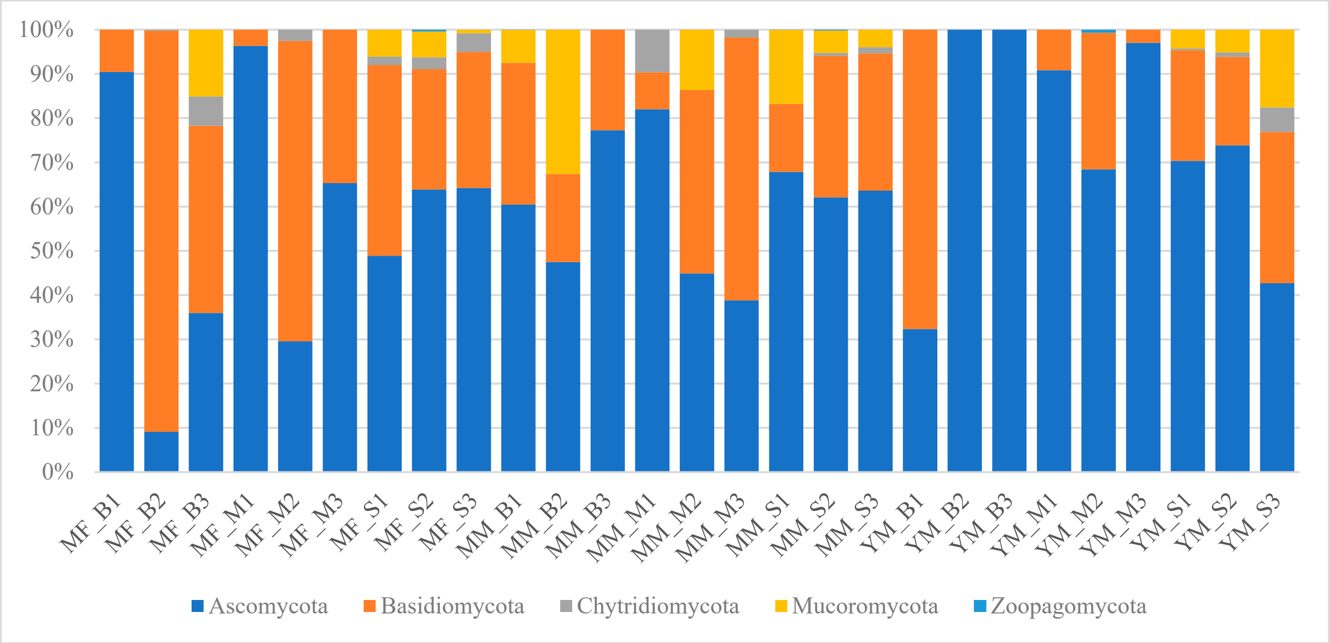

Figure S3. Community composition (phylum level) of soil fungi in this study. Mean relative abundance of three samples in each sampling site are exhibited. MF: mudflat; YM: young mangrove; MM: mature mangrove; S: surface (0-5 cm depth); M: middle (10-15 cm depth); B: bottom (20-25 cm depth).

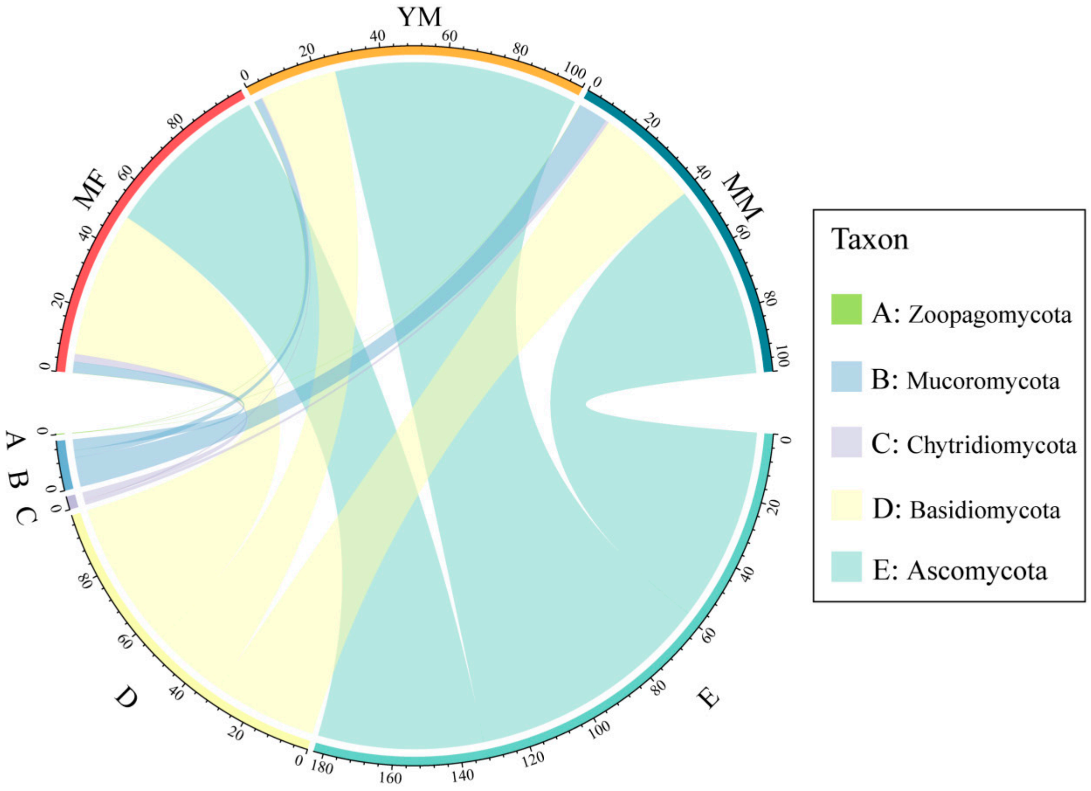

Figure S4. Community composition (phylum level) of soil fungi across different restoration stages. MF: mudflat; YM: young mangrove; MM: mature mangrove.

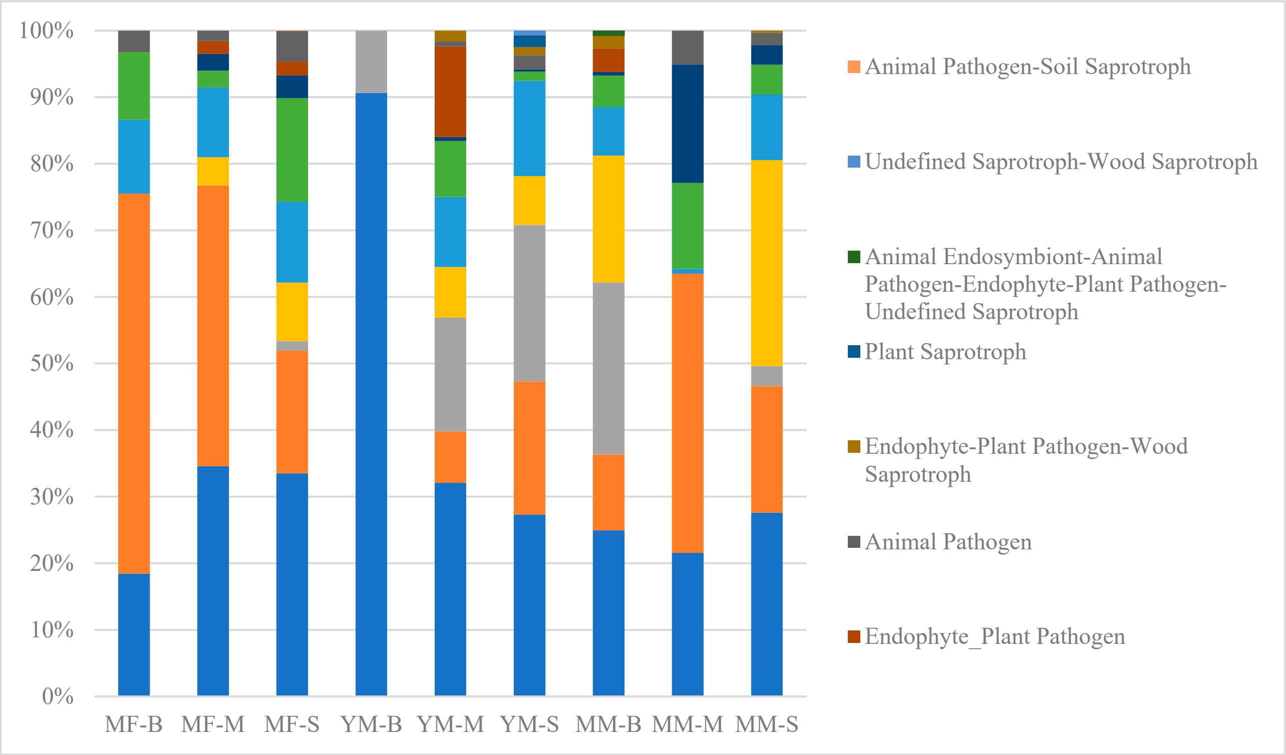

Figure S5. Relative abundance of functional guilds of the soil fungi. MF: mudflat; YM: young mangrove; MM: mature mangrove; S: surface (0-5 cm depth); M: middle (10-15 cm depth); B: bottom (20-25 cm depth).

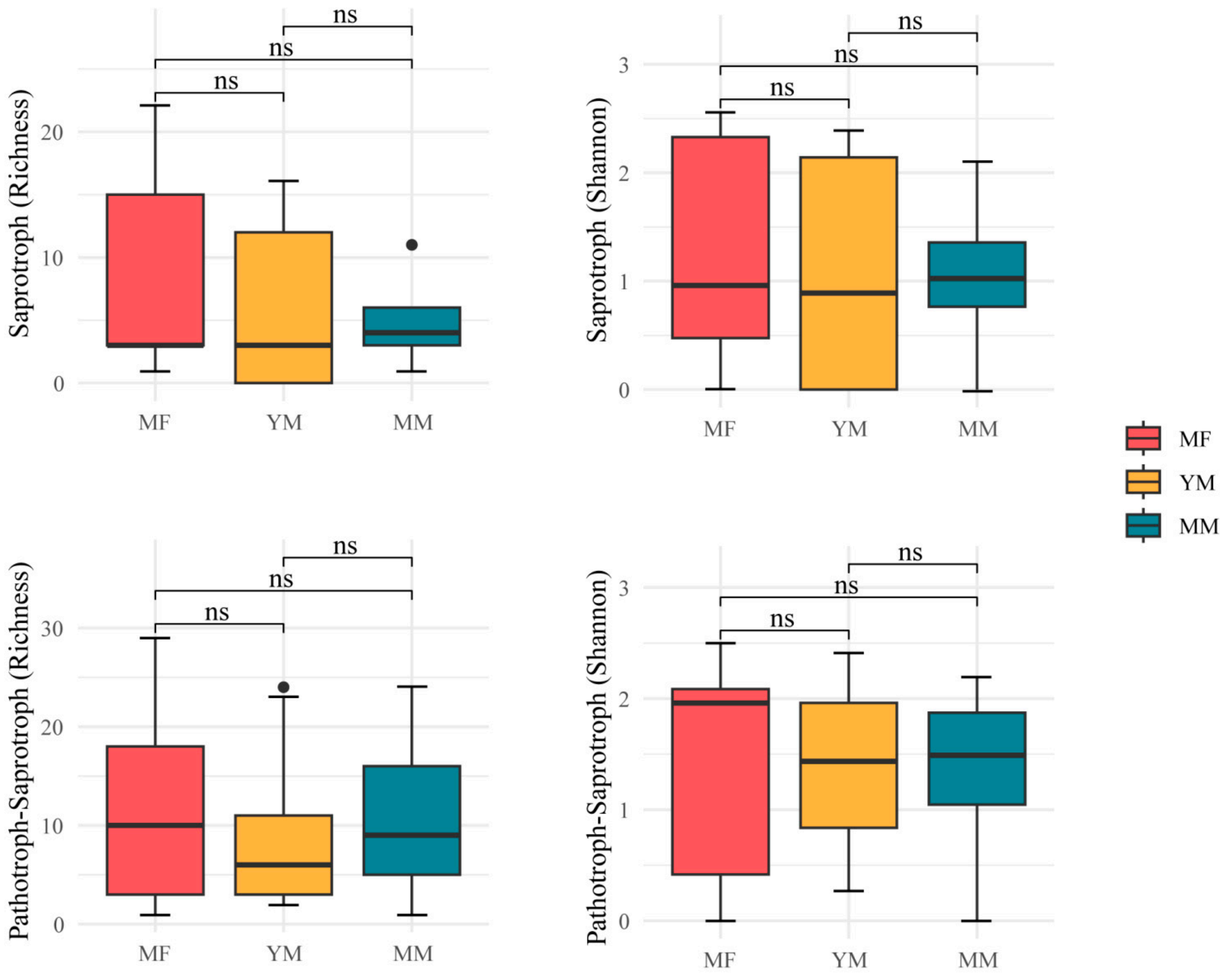

Figure S6. Alpha (α)-diversity of the most abundant trophic mode in mangrove restoration soils. MF: mudflat; YM: young mangrove; MM: mature mangrove.
